# miR-27a expression during inflammatory organ injury associated with ARDS and a novel tissue-specific knockout model

**DOI:** 10.1016/j.gendis.2025.101966

**Published:** 2025-12-06

**Authors:** Jieun Kim, Synthea Horton, Oleg D. Makarevich, Yan Levitsky, Thu Thien Tran, In Hyuk Bang, Xiangsheng Huang, Xuebo Chen, Katherine Figarella, Xiaoyi Yuan

**Affiliations:** a Department of Anesthesiology, Critical Care, and Pain Medicine, McGovern Medical School, University of Texas Health Science Center at Houston, Houston, TX 77030, USA; b Department of Pediatrics, McGovern Medical School, University of Texas Health Science Center at Houston, Houston, TX 77030, USA

Acute respiratory distress syndrome (ARDS) is a life-threatening condition with high morbidity and mortality, particularly in patients requiring surgery or intensive care.^1^ A hallmark of ARDS is acute pulmonary inflammation that progresses to systemic inflammation. MicroRNAs (miRNAs) are integral regulators of gene expression, and their dysregulation in immune cells is intricately linked to inflammatory responses.^2^^,^^3^ Among them, miR-27a has garnered attention for roles in oncogenesis, chemoresistance, and inflammation.^4^ However, their expression pattern across different tissues and immune cell populations during ARDS-associated organ injury remains underexplored, and genetic tools to study their function *in vivo* are lacking. Here, we profiled miR-27a-3p and miR-27a-5p and demonstrated that miR-27a-3p had higher expression than miR-27a-5p in most organs. However, this ratio was reversed in myeloid-derived cells, where miR-27a-5p was particularly enriched. We further examined their regulation during endotoxin-induced lung injury and found that miR-27a-5p is strongly induced under inflammatory stress in both pulmonary and non-pulmonary organs, suggesting a role in systemic immune regulation. To investigate its tissue-specific involvement, we generated a conditional knockout mouse line enabling myeloid-specific deletion of miR-27a. It resulted in altered hematopoiesis and elevated basal cytokine expression, underscoring the contribution of myeloid-derived miR-27a to inflammatory homeostasis.

During microRNA biogenesis, precursor miRNAs undergo processing to yield two mature miRNAs, miR-5p and miR-3p; typically, one strand is stabilized while the complementary strand is degraded. Notably, the co-existence of both miRNA species has been increasingly documented, suggesting a potentially significant interaction. Thus, we systematically investigated the co-expression of miR-27a-5p and miR-27a-3p in various human cell lines selected to represent major organ systems and cellular types using quantitative PCR. Our results revealed that miR-27a-3p was consistently more abundant than miR-27a-5p across diverse cell lines; however, their expression patterns demonstrated remarkable similarities (Fig. 1A). Notably, both miR-27a-3p and miR-27a-5p were most abundant in neutrophilic promyelocytes (HL-60) and least abundant in the T lymphocyte line (Jurkat). To further examine these expression levels *in vivo*, we harvested various organ compartments from mice. We found that miR-27a-3p was most abundant in the heart, with the lowest levels found in the spleen (Fig. 1B). Conversely, a remarkable increase in the expression of miR-27a-5p was observed in differentiated T cells, particularly in regulatory T cells; however, minimal expression levels were detected in the lung and small intestine. Additionally, we assessed the expression profiles of miR-27a-5p and miR-27a-3p in myeloid lineage cells, revealing the highest expression levels in neutrophils (Fig. 1C). Interestingly, we observed a shift in the ratio of miR-27a-3p and miR-27a-5p within these cells, contrasting with the expression pattern seen in other cell types. Finally, these findings were validated by the published database GSE21630 (Fig. S1A and S1B; Table S1).

**Figure 1:**
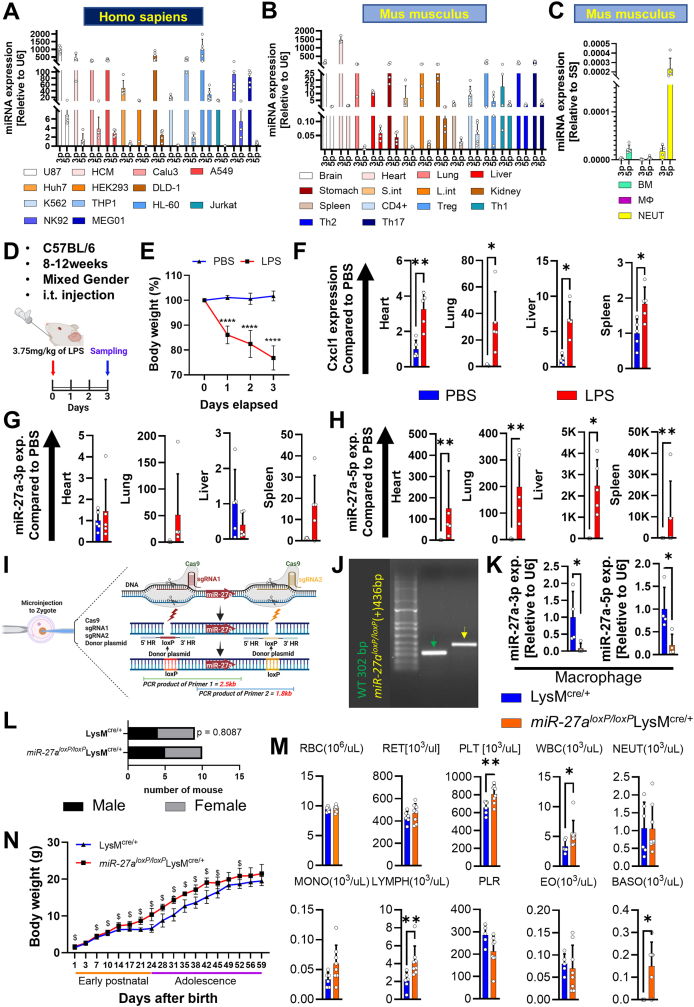
Exploration of the distinct expression profiles of miR-27a-5p and miR-27a-3p in the inflammatory organ injury associated with acute respiratory distress syndrome and characterization of *miR-27a*^*loxP/loxP*^ LysM^cre/+^ mice. **(A)** Abundance of miR-27a-5p and miR-27a-3p transcripts in human cells, which represent major organs, was quantified by quantitative PCR (*n* = 4–6). The expression level was adjusted relative to U6 snoRNA. **(B)** Quantification of miR-27a-5p and miR-27a-3p transcription levels in different organs harvested from C57BL/6J mice was performed via quantitative PCR (*n* = 3–6). The transcript level was adjusted relative to U6 snoRNA. **(C)** Transcript level of miR-27a-5p and miR-27a-3p in isolated bone marrow-derived cells (BM) from femur, isolated macrophage (Mϕ) from bronchoalveolar lavage fluid (BALF), and isolated neutrophil (NEUT) from peripheral blood were quantified by quantitative PCR (*n* = 5). Data were adjusted relative to 5S. **(D)** Illustration of the protocols. Mice received an injection of lipopolysaccharide (3.75 mg/kg of LPS, intrathecal (i.t.) injection), while the control group was given an equivalent volume of phosphate-buffered saline (PBS, i.t.). The indicating organs were collected on day 3. **(E)** Body weight measurements were taken over the study duration (*n* = 9/PBS group and *n* = 11/LPS group). Each mouse was assigned for the purpose of analyzing BALF or quantitative PCR. **(F)** Induction of CXC motif chemokine ligand 1 (Cxcl1) in the PBS and LPS groups was determined by quantitative PCR (*n* = 4 or 5/group). **(G, H)** miR-27a-3p and miR-27a-5p expression across organs in the PBS and LPS groups was quantified by quantitative PCR (*n* = 5/group). **(I)** Schematic illustration of the strategy to insert a loxP sequence into the miR-27a locus. Briefly, the target site of DNA, which is micro-injected into a fertilized mouse egg, undergoes a double-strand break caused by gRNA and Cas9 complex. The donor plasmid carrying the loxP sequence is the template for homologous recombination repair, and it recombines with the target site. **(J)** Genotyping of transgenic mice obtained through CRISPR/Cas9 targeting of the miR-27a gene. Each lane represents a genotyping result from a different mouse. PCR product size is 302 bp in the wild-type mouse (Green-arrow). The genotyping result of *miR-27a*^*loxP/loxP*^(+) showed 436 bp (Yellow-arrow). **(K)** Quantitative PCR of miR-27a-3p and miR-27a-5p transcripts in isolated macrophages of *miR-27a*^*loxP/loxP*^ LysM^cre/+^ compared with LysM^cre/+^ (*n* = 5/group). **(L)** Frequency distribution of the gender ratio. Each of the bars on that graph represents the number of litters (*n* = 9 or 10/group). **(M)** Hematological parameters. Each data point represents one animal. All graphs represent mean ± standard error of the mean. ∗*P* < 0.05 and ∗∗*P* < 0.01 by student’s *t*-test. S.int, small intestine; L.int, large intestine; CD4^+^, naïve T cells; Treg, regulatory T cell; Th1, T helper type 1; Th2, T helper type2; Th17, T helper type 17; RBC, red blood cell; RET, reticulocyte; PLT, platelet; WBC, white blood cell; NEUT, neutrophil; LYMPH, lymphocyte; PLR, platelet-to-lymphocyte ratio; MONO, monocyte; EO, eosinophil; BASO, basophil. **(N)** Growth chart reflecting the body weight of LysM^cre/+^ and *miR-27a*^*loxP/loxP*^ LysM^cre/+^ mice (*n* = 9 or 10/group). ^$^*P* < 0.05 by multiple unpaired *t*-test.

After establishing the baseline expression patterns of miR-27a-3p and miR-27a-5p across various organs and cell types, we sought to investigate how their expression fluctuates during septic ARDS-induced inflammatory organ injury. To induce this inflammation, we administered 3.75 mg/kg of lipopolysaccharide (LPS) or phosphate-buffered saline to 8-to-12-week-old C57BL/6J mice and collected vital organs after confirming sufficient body weight reduction (Fig. 1D and E). Consistent with previous publication,^5^ the severity of the disease model was indicated by elevated total protein, albumin levels, and proinflammatory cytokines in bronchoalveolar lavage fluid, highlighting the presence of lung inflammation (Fig. S2A). Recognizing that ARDS encompasses systemic inflammation and multiorgan dysfunction rather than a strictly pulmonary process, we assessed levels of CXC motif chemokine ligand 1 (Cxcl1), a pivotal player in inflammatory processes. We observed a notable increase in Cxcl1 expression across the heart, lung, liver, and spleen (Fig. 1F), suggesting heightened inflammation in those organs. Next, we analyzed the transcriptional induction of miR-27a-3p and miR-27a-5p in response to the inflammatory challenge. miR-27a-3p exhibited a trend towards increased expression in the heart, lung, and spleen in response to LPS, although these changes were not statistically significant (Fig. 1G). Notably, miR-27a-5p exhibited a striking induction in the LPS-treated group, with an increase of approximately 100-fold (Fig. 1H). Our observations suggest that miR-27a-5p is significantly induced during organ inflammation and might play critical roles in the inflammatory response.

To establish an animal model to study the functions of miR-27a *in vivo*, we developed a novel transgenic mouse line utilizing CRISPR/Cas9 technology (Fig. 1I). The gRNA targeting the mouse miR-27a gene, along with the donor vector containing LoxP sites and Cas9 mRNA, was co-injected into fertilized mouse eggs to produce offspring with targeted conditional deletions. F0 founder animals were identified through PCR and sequence analysis, were subsequently bred to C57BL/6J mice to achieve germline transmission, generating F1 animals (*miR-27a*^*loxP/loxP*^). The PCR genotyping procedure employing primer pairs F3 and R3 (primer sequence described in the methods) facilitated the amplification of 302-bp and 436-bp bands, reflecting the wild-type and mutant alleles, respectively (Fig. 1J). By crossing these floxed mice with myeloid-derived Cre-expressing mice (LysM^cre/+^), we aimed for the selective deletion of the floxed region, facilitating the inactivation of miR-27a specifically in myeloid-derived cells (*miR-27a*^*loxP/loxP*^/LysM^cre^), while maintaining intact in other tissues. To verify successful deletion of miR-27a in myeloid-derived cells, we isolated macrophages and demonstrated a significant reduction of miR-27a in *miR-27a*^*loxP/loxP*^/LysM^cre^ mice, while the expression levels remained unaltered in most of the major organs (Fig. 1K; Fig. S3A). Interestingly, we also noted a reduction of miR-27a in the spleen, potentially attributable to its diverse composition of dendritic and myeloid cell subsets.

After confirming successful deletion of miR-27a, we next investigated the baseline phenotype of *miR-27a*^*loxP/loxP*^/LysM^cre/+^. To evaluate reproductive performance and growth rates in LysM^cre/+^ and *miR-27a*^*loxP/loxP*^/LysM^cre/+^ mice, we tracked the litter from these mice and conducted a Chi-square test to examine potential associations between the depletion of miR-27a in myeloid lineage cells and the sex of the offspring. Our analysis did not yield any significant effect of miR-27a knockout on the sex of the offspring (Fig. 1L). Growth velocities diverged between the two groups during the early postnatal phase and extended into mid-adolescence (∼ postnatal day 46), although growth rates eventually equalized during late adolescence (Fig. 1N). Subsequently, we evaluated the impact of myeloid-specific miR-27a deletion on basal proinflammatory signaling across organs by detecting Cxcl1. Surprisingly, the expression level of Cxcl1 was elevated in the lung of *miR-27a*^*loxP/loxP*^/LysM^cre/+^ compared with corresponding control samples, suggesting potential baseline inflammation (Fig. S3B). Finally, we further analyzed the hematological parameters of both LysM^cre/+^ and *miR-27a*^*loxP/loxP*^/LysM^cre/+^ mice (Fig. 1M). Notably, *miR-27a*^*loxP/loxP*^/LysM^cre/+^ mice exhibited significantly elevated levels of platelets, white blood cells, lymphocytes, and basophils. These findings suggest that myeloid-derived miR-27a plays a pivotal role in modulating immune responses and hematological processes.

In conclusion, we investigated the expression of miR-27a across multiple cell types and organs and characterized the inflammatory response and miR-27a expression levels across different organ systems during endotoxin-induced lung injury. Moreover, we developed a novel mouse model using CRISPR/Cas9 technology to allow tissue/cell-specific deletion of miR-27a *in vivo*. Our results demonstrated intriguing abnormalities in hematopoiesis under homeostatic conditions during myeloid deletion of miR-27a, implicating potential systemic functional outcomes.

## CRediT authorship contribution statement

**Jieun Kim:** Writing – review & editing, Writing – original draft, Formal analysis, Data curation. **Synthea Horton:** Methodology, Investigation. **Oleg D. Makarevich:** Writing – review & editing, Methodology, Investigation. **Yan Levitsky:** Writing – review & editing, Methodology, Data curation. **Thu Thien Tran:** Writing – review & editing, Validation. **In Hyuk Bang:** Writing – review & editing, Validation. **Xiangsheng Huang:** Writing – review & editing, Investigation. **Xuebo Chen:** Resources, Methodology. **Katherine Figarella:** Writing – review & editing, Validation. **Xiaoyi Yuan:** Writing – review & editing, Supervision, Funding acquisition, Conceptualization.

## Ethics declaration

Animal procedures were approved by the Animal Welfare Committee (AWC) at the University of Texas Health Science Center (UTHealth) at Houston (AWC-23-0058). Informed consent was obtained from all the subjects involved in the study.

## Funding

This work is supported by the Parker B. Francis Fellowship, American Lung Association Catalyst Award and the National Institutes of Health (Bethesda, Maryland) (No. R01HL155950 to Xiaoyi Yuan).

## Conflict of interests

The authors declared no conflict of interests.
