# Supplementary material for: miR-27a expression during inflammatory organ injury associated with ARDS and a novel tissue-specific knockout model
Source: Genes Dis. 2025 Dec 6;13(5):101966. doi: 10.1016/j.gendis.2025.101966 (PMC13127179; doi:10.1016/j.gendis.2025.101966)
Supplement: Multimedia component 1 [file mmc1.docx]

**miR-27a expression during inflammatory organ injury associated with ARDS and a novel tissue-specific knockout model**

Jieun Kim^1^, Synthea Horton^1^, Oleg D. Makarevich^1^, Yan Levitsky^1^, Thu Thien Tran^2^, In Hyuk Bang^1^, Xiangsheng Huang^1^, Xuebo Chen^1^, Katherine Figarella^1^, Xiaoyi Yuan^1^

**Affiliations:**

^1^Department of Anesthesiology, Critical Care, and Pain Medicine, McGovern Medical School, University of Texas Health Science Center at Houston, Houston TX 77030, USA

^2^Department of Pediatrics, McGovern Medical School, University of Texas Health Science Center at Houston, Houston TX 77030, USA

**Correspondence:**

Xiaoyi Yuan, Ph.D.

Department of Anesthesiology, Critical Care, and Pain Medicine

The University of Texas Health Science Center at Houston, McGovern Medical School

6431 Fannin Street

Houston, TX 77030, USA

Phone: 713 500 6307

Email: Xiaoyi.yuan@uth.tmc.edu

**Materials and Methods**

**Animals.** Animal procedures were approved by the Institutional Animal Care and Use Committee at the University of Texas Health Science Center (UTHealth) at Houston (AWC-23-0058). C57BL/6J (wild-type; WT) and LysM^cre/+^ mice were acquired from Jackson Laboratories (Bar Harbor, ME). All mice were housed and bred in a specific pathogen-free facility at the Center for Laboratory Animal Medicine and Care at the UTHealth. Both gender mice, aged between 8 and 12 weeks, were used in all experiments. In order to facilitate tissue-specific deletion of miR-27a, *miR-27a^loxP/loxP^* mice were bred with B6.129P2-Lyz2^tm1(cre)Ifo^/J mice (LysM^cre/+^) to generate *miR-27a^loxP/loxP^* LysM^cre/+^ mice.

**Generation of transgenic miR-27a mutant mice.** To generate a miR-27a deficient mouse line, the fertilized mouse eggs were co-injected with guide RNAs (gRNA) targeting the mouse miR-27a gene, a donor vector containing loxP sites, and Cas9 mRNA to produce offspring with a targeted conditional knockout. Two individual single gRNA targeting pre-miR-27a were designed: gRNA 1 binding site: 5’- AGACAGACATCCTTGGCGACAGG-3', and gRNA2 binding site: 5’- GGGTGGGCAGCAGCGGCCTGAGG-3'. Those sequences are matched to the reverse strand of the gene. The T7 promoter and the scaffold were added using overlapping PCR with the following primers listed from 5’ to 3’: 5’ forward primer (F1): 5’-GACCTATCACTGAAGACCCTGATG-3’, 3’loxP reverse primer (R1): 5’-GTGGATTCGGACCAGTCTGA-3’, 5’loxP forward primer (F2): 5’-ACGTAAACGGCCACAAGTTC-3’, 3’arm reverse primer (R2): 5’-TAGTTCATGGTGCGTTCCTTTG-3’. 5’Sequence primer (F6): 5’-GCTCCAATCTCACTGTCTCTTCT-3’ and 3’Sequence primer (R6): 5’-ACTAATTACCCCATTGTTTCCAGGT-3’ are used for sequencing confirmation. Genotyping was performed using the following method: PCR product was carried out in 50ul volume for 33 cycles under standard conditions, with Forward primer (F3): 5’-TGATGCCAGTCACAAATCACATTG-3’ and Reverse primer (R3): 5’-TCAGTAGGCACGGGAGGCGGAG-3’added to each reaction. Taq DNA polymerase utilized was Long Amp Taq DNA polymerase (NEB, Cat No. #M0323V).

**LPS model.** We performed a lipopolysaccharide (LPS) injection procedure to establish an endotoxin-induced organ injury model. A total of 20 mice were randomly divided into two groups (n=-9-11/group) as follows: i) Control and ii) LPS. Mice were anesthetized with isoflurane. LPS group mice were injected intratracheally with 3.75 mg/kg LPS (from E. coli (O111:B4), Millipore Sigma, Cat No. #L4391). The control group received the same volume of PBS. All mice were euthanized 3 days after LPS treatment, and the organs and bronchoalveolar lavage fluid (BALF) were collected for subsequent analysis.

**Cell lines.** HEK293, T84, A549, Calu3, DLD-1, HK-2, Huh7, HL-60, K562, MEG-01, NK92, Jurkat, U87, HCT-8 and THP1 cells were acquired from ATCC and maintained in accordance with ATCC’s guidelines. Human cardiomyocytes (HCM) were acquired from ScienCell (Cat No. #6200) and maintained in accordance with manufacturer’s suggested culture condition.

**Isolation of bone marrow immune cells, blood neutrophils, and macrophage.** To isolate bone marrow from the femurs of mice, 10 mL of RPMI 1640 medium (Cat No. #10-040-CV, Corning) supplemented with 10% FBS and 1% penicillin-streptomycin is used to flush out the bone marrow with a 25-gauge needle attached to a syringe. The resuspended marrow sample is then strained through a 70 µm cell strainer, ensuring any residual marrow is rinsed from the plunger end. After centrifugation, an ACK lysing buffer (Cat No. #118-156-101, Quality Biological) was applied to the cell pellet on ice for 10 minutes to lyse red blood cells, followed by the collection of bone marrow immune cells after removing the supernatant. Neutrophils were isolated from the blood of 8-12 week-old C57BL6J mice. Following the recommended protocol provided by the manufacturer, neutrophils were isolated from the blood of 8-12 week-old C57BL6J mice using EasySep™ Mouse Neutrophil Enrichment Kit (Cat No. #19762, STEMCELL Technologies). To isolate alveolar macrophage, BALF is obtained by inserting a catheter into the trachea of terminally anesthetized mice, followed by the instillation of sterile PBS into the bronchioles. The retrieved fluid was then centrifuged at 800 xg for 10 minutes to harvest alveolar macrophage for subsequent analysis. The cell pellet was lysed with a QIAzol lysis reagent (Cat No. # 79306, QIAGEN) reagent for RNA isolation.

**Sorting naïve CD4 T cells and T cell differentiation.** naïve CD4 T cells were created by filtering the spleen or mesenteric lymph node through a 100mm cell strainer. Erythrocytes were lysed after a 5 minutes exposure to a ACK lysing reagent (Cat No. #118-156-101, Quality Biological). The resulting cell suspension was then collected by centrifugation and resuspended in a complete medium. T cell differentiation to Th1/Th2/Th17/Treg conditions were done following condition; supplement the culture media with cytokines and blocking antibodies accordingly. Th1: 15 ng/ml rmIL-12, 30 U/ml hIL-2, and 5,000 ng/ml 11B11.Th2: 10 ng/ml rmIL-4, 30 U/ml hIL-2, 2,000 ng/ml soluble 37.51, and 5,000 ng/ml XMG1.2. Treg: 15 ng/ml hTGFβ, 30 U/ml hIL-2, 5,000 ng/ml XMG1.2, and 5,000 ng/ml 11B11. Th17: 20 ng/ml rmIL-6, 3 ng/ml hTGFβ, 5,000 ng/ml XMG1.2, and 5,000 ng/ml 11B11. Cells were incubated for 72hours and then rinsed with PBS. Following centrifugation, the cell pellet was lysed using QIAzol lysis reagent (Cat No. # 79306, QIAGEN) to facilitate RNA isolation.

**Quantitative Real-time Polymerase Chain Reaction.** Total RNA was isolated from cultured cells and mouse tissues using a QIAzol lysis reagent (QIAGEN, Cat No. # 79306) according to the manufacturer’s instructions. A High-Capacity cDNA RT kit (Applied Biosystems, Thermo Fisher Scientific) was used to perform reverse transcription on 400ng of total RNA. TaqMan real-time PCR assay was conducted to measure interleukin-6 (Il6), chemokine CXC motif ligand 1 (Cxcl1), and Actb (internal control). TaqMan™ Gene Expression Assay (FAM): Actb (Cat No. # 4331182, Assay ID: Mm02619580_g1); Il6 (Cat No. # 331182, Assay ID: Mm00434228_m1); Cxcl1 (Cat No. # 4331182, Assay ID: Mm04207460_m1). For miRNA detection, quantitative PCR (qPCR) was carried out using SYBR Green MicroRNA Assay in the qPCR system. Briefly, with 400ng of total RNA, reverse transcription was executed using the miRCURY LNA RT kit (Cat No. #339340, QIAGEN), employing primers for miR-27a-3p, miR-27a-5p, U6 snoRNA, and 5S (internal control) primers on a Bio-Rad T100 Thermal Cycler. And then, the qPCR product was amplified using miRCURY LNA miRNA PCR Assay plus the amfiSure qGreen q-PCR Master Mix(2X) without ROX (Cat No. #Q5600-005, GenDEPOT) on Bio-Rad CFX384 real-time system. Target gene expression levels were calculated using the 2−∆∆Ct method, adjusted by the internal control for normalization. QIAGEN™ MicroRNA Assay: miR-27a-3p (Cat No. # 339306, GeneGlobe ID: YP00206038); miR-27a-5p (Cat No. # 339306, GeneGlobe ID: YP00206021); U6 snRNA (v2) (Cat No. # 339306, GeneGlobe ID: YP02119464); 5S (Cat No. # 339306, GeneGlobe ID: YP00206021).

**Biomarker measurement.** The total protein concentration in BALF was determined using a colorimetric assay (Bio-Rad, Cat No. #5000205, Quick Start Bradford 1x Dye Reagent). Additionally, mouse CXCL1 (R&D SYSTEMS, Cat No. #DY453) and IL6 (R&D SYSTEMS, Cat No. #DY406) levels were quantified using commercially available ELISA kits.

**Histopathological analysis.** Once harvested, tissues were immersed in 10% formaldehyde for 24 to 48 hours and then underwent a dehydration process using a gradient of ethanol (ranging from 70 % to 100 %) and xylene before being embedded in paraffin. Five-micrometer sections of the tissue were then cut and stained with hematoxylin and eosin (H&E). Images were captured from the slides using a Leica microscope (DMC 2500LED).

**Gene expression data in the GEO database.** Gene expression data were sourced from the Gene Expression Omnibus (GEO) database (<http://www.ncbi.nim.nih.gov/geo/>). For analysis of miR-27a-3p and miR-27a-5p expression, we utilized the GSE21630 dataset, which features small RNA expression profiles from 27 well-characterized cell types in the mouse immune system, along with hematopoietic progenitor cells, embryonic stem cells, and 12 distinct tissues. This dataset comprises both biological and technical replicates for three of cell types.

**Statistical analysis.** All data are presented as mean ± standard error of the mean (24). For data conforming to normal distribution and equal variances, parametric two-sample unpaired t-tests were employed for comparison. In contrast, data that were not normally distributed were analyzed with non-parametric two-sample unpaired t-tests and Mann-Whitney rank-sum tests. A comparison involving three or more groups was performed using one-way ANOVA, with corrections applied for multiple comparisons. The figure legend contains comprehensive information about the statistical analyses conducted for each experiment. These analyses were executed with the assistance of GraphPad Prism software.

**Supplementary Figure**


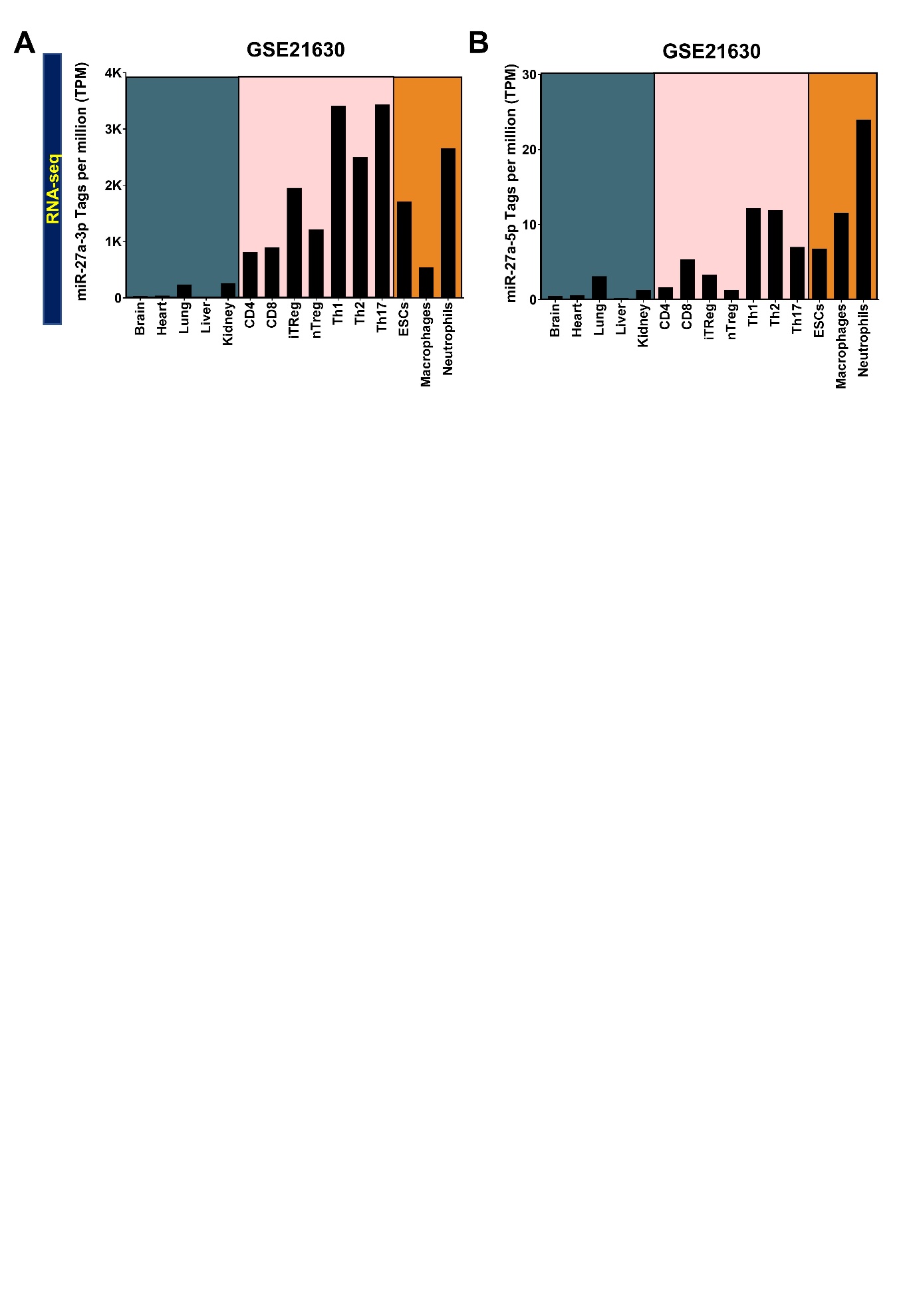


**Supplementary Figure 1.** Validation of miR-27a-3p and miR-27a-5p profile. **(A-B)** GSE21630 showing diversity in the expression of miR-27a-3p and miR-27a-5p in cells derived mouse. Dark green-colored indicated major organs. Pink-colored indicated lymphoid lineage cells. Orange-colored indicated myeloid lineage cells. All graphs represent mean ± SEM.


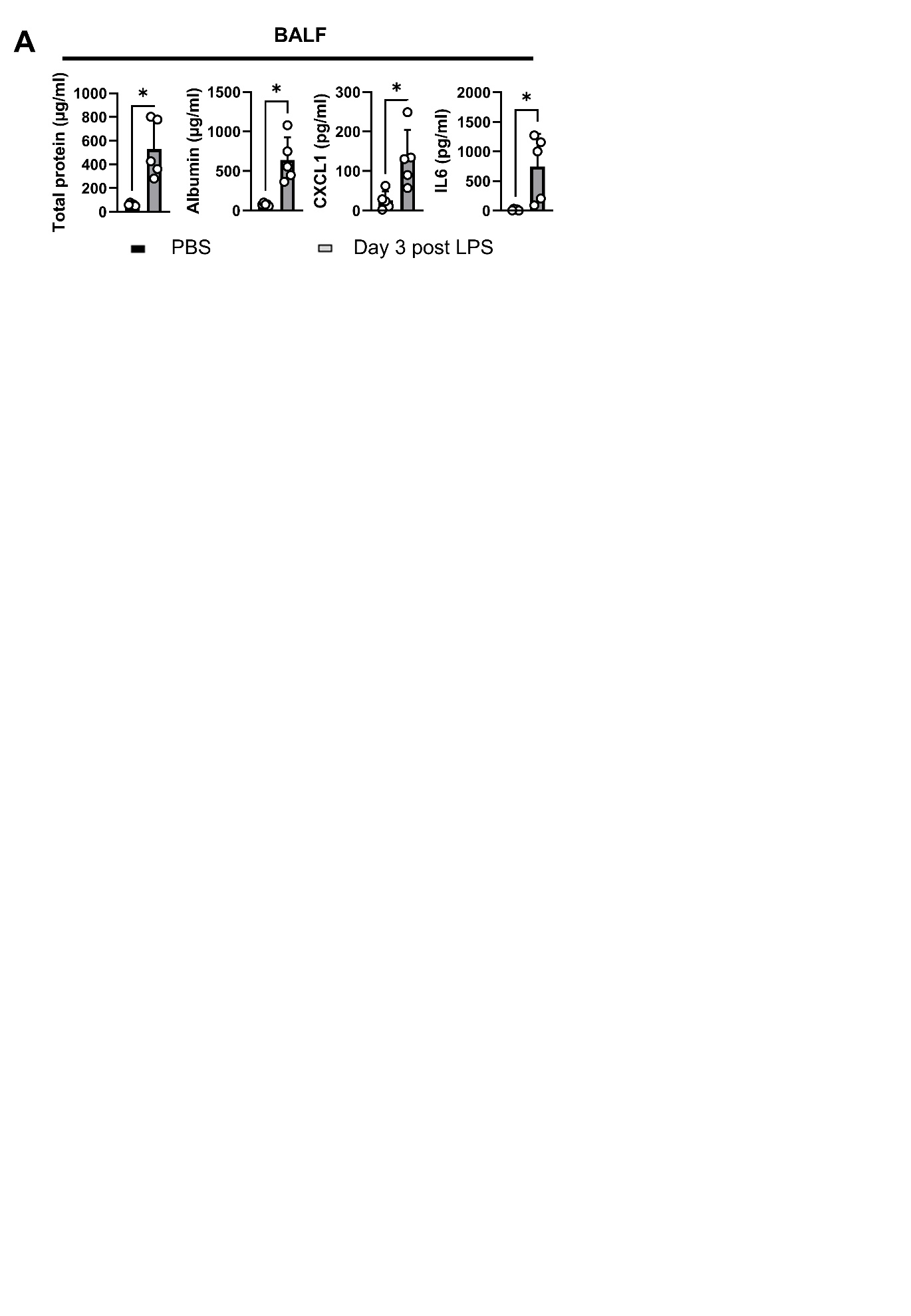


**Supplementary Figure 2.** Evaluation of lipopolysaccharide (LPS)-induced lung injuries and inflammation mediators in BALF **(A)** Protein concentration was measured in BALF with Bradford Assay (n = 5/group). Albumin level was measured in BALF with Albumin ELISA (n = 5/group). The concentration of pro-inflammatory mediators in BALF was determined by ELISA (n = 5/group). All graphs represent mean ± SEM. *P < 0.05 by student’s t-test.


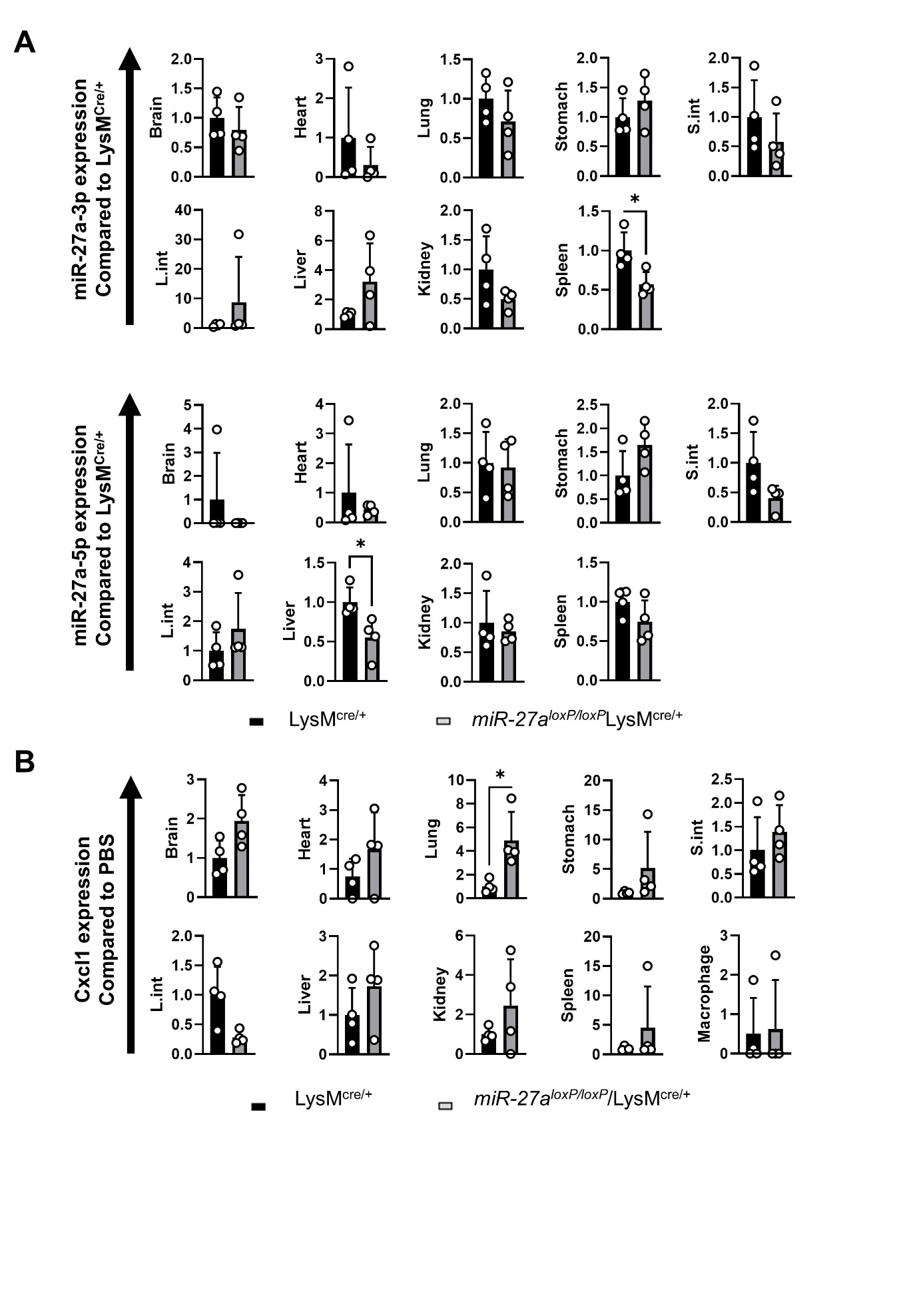


**Supplementary Figure 3.** Expression pattern of Cxcl-1, miR-27a-3p, and mR-27a-5p across different organs in LPS-induced ARDS. **(A)** Induction of chemokine CXC motif ligand 1 (Cxcl1) in PBS and LPS group was determined by qPCR (n = 4-5/group). (**B**) miR-27a-3p and miR-27a-5p expression across organs in phosphate buffered saline (PBS) and LPS group was quantified by qPCR (n = 5/group). All graphs represent mean ± SEM. *P < 0.05 and **P < 0.01 by student’s t-test.
